# The growth of *Escherichia coli* cultures under the influence of pheomelanin nanoparticles and a chelant agent in the presence of light

**DOI:** 10.1371/journal.pone.0265277

**Published:** 2022-03-11

**Authors:** Denisse Fuentes-López, Daniel Ortega-Zambrano, María Antonieta Fernández-Herrera, Hilda Mercado-Uribe

**Affiliations:** 1 Centro de Investigación y de Estudios Avanzados, del Instituto Politécnico Nacional Unidad Monterrey, Apodaca, Nuevo León, México; 2 Departamento de Física Aplicada, Centro de Investigación y de Estudios Avanzados, del Instituto Politécnico Nacional Unidad Mérida, Mérida, Yucatán, México; United Arab Emirates University, UNITED ARAB EMIRATES

## Abstract

Growing concern of antibiotic resistance has increased research efforts to find nonspecific treatments to inhibit pathogenic microorganisms. In this regard, photodynamic inactivation is a promising method. It is based on the excitation of a photosensitizer molecule (PS) with UV-Vis radiation to produce reactive oxygen species. The high reactivity of such species nearby the PS leads to oxidation of bacterial cell walls, lipid membranes (lipid peroxidation), enzymes, and nucleic acids, eventually producing cell death. In the last decade, many studies have been carried out with different photosensitizers to suppress the growth of bacteria, fungi, viruses, and malignant tumors. Here, our main motivation is to employ pheomelanin nanoparticles as sensitizers for inhibiting the growth of the Gram-negative bacteria *E*. *coli*, exposed to blue and UVA radiation. In order to perform our experiments, we synthesized pheomelanin nanoparticles from L-DOPA and L-cysteine through an oxidation process. We carried out experiments at different particle concentrations and different energy fluences. We found that cultures exposed to UVA at 166 μg/mL and 270 J/cm^2^, in conjunction with ethylenediaminetetraacetic acid (EDTA) as an enhancer, decreased in the viable count 5 log10. Different reactive oxygen species (singlet oxygen, hydroxyl radicals, and peroxynitrates) were detected using different procedures. Our results suggest that the method reported here is effective against *E*. *coli*, which could encourage further investigations in other type of bacteria.

## Introduction

Photodynamic inactivation (PDI) has emerged as a valuable method to inhibit pathogen microorganisms and reduce infectious diseases in an era where the resistance of bacteria to antibiotics is an important medical issue. Three components in PDI are needed: a photosensitizer dye (PS), light (of a given wavelength to excite the PS), and molecular oxygen. Light excites the PS, which goes from its ground singlet state to an excited one. Then, the PS returns to the ground singlet state releasing part of the absorbed energy by fluorescence or heat. However, the PS may decay to a state of lower energy, the excited triplet state, by an intersystem crossing process. Finally, the PS returns to the ground state through phosphorescence or by generating reactive oxygen species (ROS), either by charge transfer such as hydrogen peroxide and hydroxyl radicals to a surrounding substrate (type I process) or energy transfer directly (type II process) to the ground state of molecular oxygen (^3^O_2_). The last mechanism results in the formation of singlet oxygen (^1^O_2_), one of the leading players in the phototoxic reaction [1, 2]. The high reactivity of ROS and ^1^O_2_ induces oxidative reactions nearby the PS. Oxidation of bacterial cell walls, lipid membranes (lipid peroxidation), enzymes, and nucleic acids, eventually leads to cell death [3]. This mechanism is advantageous concerning antibiotic action, since PDI acts not upon a specific target but on several ones [4]. Such multi-specificity may imply a relatively low bacterial resistance. Gram-negative bacteria, like *E*. *coli*, have two lipid bilayers instead of one, separated by a peptidoglycan structure. They are negatively charged and thus impermeable to anionic chemicals, and more difficult to inactivate than Gram-positive bacteria [5, 6]. For this reason, it is essential to properly select the PS. Concerning this point, there have been some efforts to photoinactivate *E*. *coli* using neutral and cationic photosensitizers, as well as a combination of both [7–9].

Melanins are complex polymers derived from the precursor dopaquinone through sequential chemical reactions [10, 11]. They are synthetized as pigments by melanosomes of mature melanocytes and are taken by dendrites to the keratinocytes in the epidermis. Melanin determines the color of the skin, eyes, and hair, and is a combination of two types of pigments: eumelanin (a dark brown to black pigment) and pheomelanin (a yellow to reddish-brown pigment) [10, 12]. The first contains nitrogen but no sulphur, the second contains both of them [13, 14]. Since pheomelanin and eumelanin are structurally distinct, they exhibit different biochemical activity. While pheomelanin is alkali-soluble, eumelanin is insoluble in almost all solvents [15, 16].

The role of melanins is still controversial [10, 12, 17]; some authors agree that eumelanin has a protective function, while pheomelanin is phototoxic due to the generation of ROS after UV exposure. It has been observed that pheomelanin generates superoxide anions and reduces some antioxidants like glutathione (GSH) [18]. In any case, the functions of melanins are usually associated with their effects produced by light. More studies are needed to understand the melanogenesis process in detail.

Although many investigations have been performed to understand the function of eumelanin, studies on pheomelanin are scarce [11, 13, 18–26]. The main reason is due to the difficulty to synthesize and characterize it. Eumelanin is commercially available, while the synthesis or laborious extraction in the laboratory is the only via for obtaining pheomelanin. For instance, Ito et al. [20, 22] worked on the synthesis process of pheomelanin mainly by an enzymatic reaction (using tyrosinase). In this work, we synthesized pheomelanin nanoparticles based on a reported method by Pyo et al. [25], who used a chemical oxidation with KMnO_4_. In our synthesis we made small modifications in the protocol. We characterized the particles using different complementary techniques: dynamic light scattering (DLS), Fourier transformed infrared spectroscopy (FTIR), X-ray diffraction analysis (XRD), energy dispersive X-ray spectroscopy (EDS) and scanning electron microscopy (SEM), see complete details in S1 File, S1–S4 Figs and S1 Table [24, 25, 27–37]. Thereafter, we investigated the inactivation effect of the pheomelanin nanoparticles in *E*. *coli* cultures subjected to blue and UVA irradiation. It is important to remark that the photodynamic inactivation pursued by the group of Pyo et al. [25] focused on HeLa cells, using UVC light and pheomelanin particles. The authors found a reduction of around 50% of viability. In another study, Yi-Cheng et al. [38] investigated the effect of Sepia and synthetic melanin in Gram-positive bacteria irradiated at 660 nm, reporting non-significant response.

**Fig 1:**
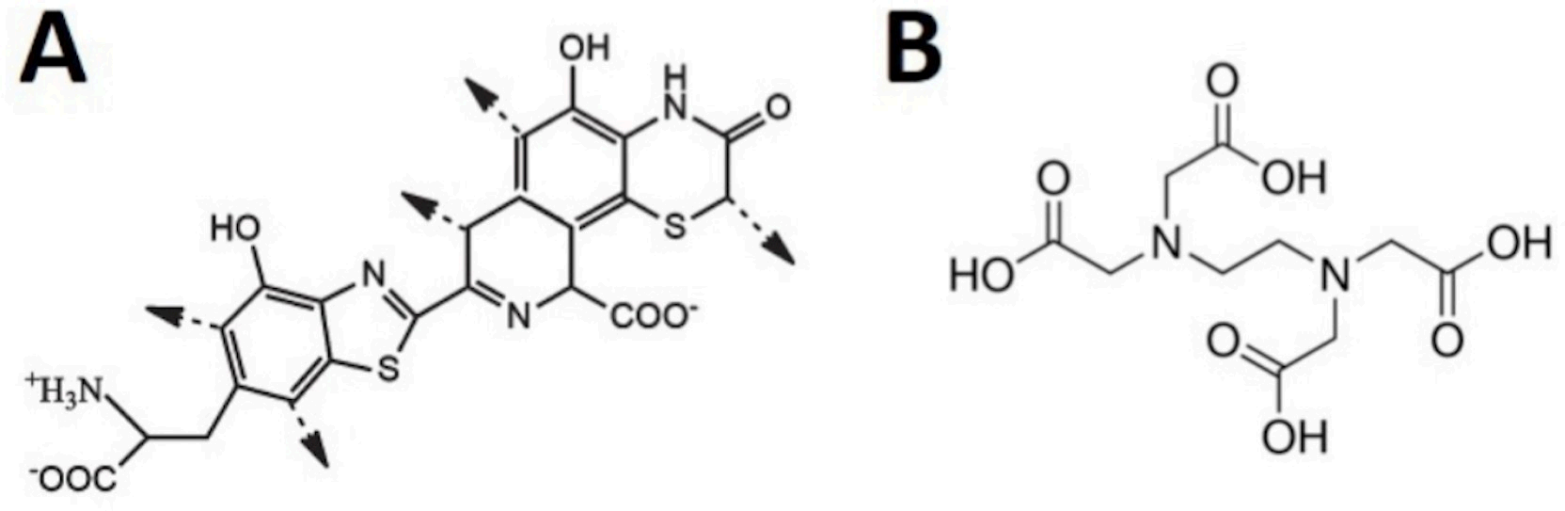
Chemical structures of (A) pheomelanin [44] and (B) EDTA [45].

We explore different energy fluences and concentrations of pheomelanin particles. We show that the most significant inactivation is obtained with UVA radiation at 270 J/cm^2^ and a concentration of 166 μg/mL. To prove if ROS and singlet oxygen were involved in the observed inactivation, we performed two experiments using molecular probes and an optical setup. In order to facilitate the action of PS’s in microorganisms, especially in Gram-negative bacteria, chelant agents like EDTA (which is a di and trivalent metal ion sequester) are commonly used [5]. Indeed, it has been proved that the treatment with a chelant agent in Gram-negative wild-type bacteria promotes the electrostatic repulsion between lipopolysaccharide components and removes divalent cations; consequently, destabilizing the structure of the cell [39]. This action facilitates the permeation of PS in the cell membrane, and when PS is activated by light, the cell is efficiently photosensitized [40–42]. Since our pheomelanin nanoparticles exhibited negative superficial charge, we also enhanced the photoinactivation efficacy adding this chelant. Using this strategy, we obtain a reduction of 5 log10 steps, which is much better than the Word Health Organization (WHO) criterion recommended in the reduction of the CFU [43]. For completeness, we show the structures of pheomelanin and EDTA, see Fig 1.

## Material and methods

As previously mentioned, the synthesis process of pheomelanin nanoparticles [25] and characterization [27–30] are carefully detailed in S1 File, S1–S4 Figs and S1 Table. Next, we describe some important experimental issues.

### Dynamic light scattering measurements

Particle size and zeta potential were determined by dynamic light scattering (DLS) using a Malvern Zetasizer NanoZS equipment [46]. A sample (1 mL) of the stock suspension was poured into a disposable polystyrene cuvette. It was equilibrated at 25°C for 120 s and exposed to a 633 nm laser. The zeta potential was evaluated in different solvents: milli-Q water, PBS and PBS+EDTA. Three independent measurements were performed, each one with twelve iterations, and the average was obtained.

### Bacterial culture preparation

*E*. *coli* K12-MG-1655 was used in this work. The strain was previously stored at -80°C in a Luria Bertani (LB) broth with 20% of glycerol. A small amount of cell material was placed in a culture tube with 2 mL of LB medium. The sample was incubated at 37°C for 24 h in an orbital shaker. Then, 200 μL of a bacteria aliquot was put into 20 mL of LB medium and left for incubation again, at 37°C and 180 r.p.m. The optical density (OD) of the suspension was adjusted to 0.3 (exponential phase) using a spectrophotometer (Multiskan GO, Thermo Scientific). Such OD corresponds to ∼ 10^8^ colony-forming units (CFU)/mL. Then, three serial dilutions were carried out as follows: 10 mL of the bacterial culture were poured in 10 mL of LB medium, and the suspension was shaken for 140 min at 180 r.p.m. Bacteria were harvested by centrifugation, washed and resuspended three times in phosphate-buffered saline (PBS) at pH 7.4 and mixed by vortexing. The first experiment consisted in exploring the direct effect of pheomelanin in the *E*. *coli* culture. Then, 100 μL of bacteria suspension were mixed with 900 μL of pheomelanin suspension in Pyrex brand culture tubes (2 mL). We used four different concentrations of this suspensions: 83, 123, 166 and 247 μg/mL. In order to increase the permeability of the outer membrane of bacteria, a second experiment was carried out using EDTA at 10 mM. 300 μL of each bacterial suspension were seeded in each well of a 96-well microplate and incubated for 30 min in dark conditions.

### Photodynamic inactivation of *E*. *coli* cultures

First, we investigated the photosensitization of *E*. *coli* bacteria with pheomelanin nanoparticles and blue light (PS+Blue Light, 450 nm) using the concentrations above described, and three fluences: 90, 180, and 270 J/cm^2^ (corresponding to exposure times of 1, 2, and 3 h). Next, we exposed the cultures to UVA radiation (PS+UVA at 375 nm), at a power of 8 mW. We chose the concentration of 166 μg/mL due to the low level of dark toxicity and 270 J/cm^2^ because the obtained photoinactivation of blue light was the best. A neutral density filter was used to maintain the same incident power coming from the light source. Exposures were performed employing a blue laser diode (Laserland), and a UVA laser (Excelsior Spectra-Physics). The area of the spot light was 0.322 cm^2^ and the irradiance was 24.8 mW/cm^2^. The variation of the temperature during the experiment (3 h) was ± 1°C. The chelant agent EDTA was used as a photoinactivation enhancer in a similar way as previously reported by other authors [5, 40, 42]. Pertinent controls (PS, light, EDTA, and PS+EDTA) were carried out. Each sample was plated in triplicate using LB medium, and the colony-forming units were counted after 48 h with a previous incubation at 37°C.

### Fluorescence assays measurements

To identify the main types of ROS generated in the photoinactivations with UVA (the case with the greatest effect), we used Singlet Oxygen Sensor Green, SOSG (Invitrogen) and hydroxyphenyl fluorescein, HPF (Invitrogen). These are probe molecules that produce fluorescence signals. SOSG reacts with ^1^O_2_ resulting in SOSG endoperoxides (SOSG-EP), which emit green fluorescence with an excitation and emission maxima around 504 and 525 nm, respectively. HPF detects selectively highly ROS. It is very sensitive to hydroxyl radicals (˙OH) and peroxynitrates (ONOO^-^) exhibiting a green fluorescence upon oxidation, with a peak at around 515 nm. To detect ^1^O_2_, we prepared a solution of SOSG (1.92 μM) diluted in methanol and water. We mixed 20 μL of a pheomelanin suspension (1.42 mg/mL), 1.8 mL of SOSG solution and 180 μL of PBS buffer. The final concentration of the suspension was 1 μM [47]. In the case of HPF, we proceeded in the same way obtaining a suspension with a final concentration of 5 μM [48]. These suspensions were exposed to a 16 mW UVA laser (Excelsior 375 Spectra-Physics) for 2 hours in polystyrene cuvettes. Fluorescence spectra were measured using a FluoroMax-4 spectrofluorometer (HORIBA Yobin Yvon). SOSG was excited at 504 nm and SOSG-EP recorded between 500 and 650 nm. The excitation of HPF was at 490 nm and the fluorescence registered around 513 nm. Fluorescence intensity was measured every 30 minutes. The excitation and emission slits were set to 5 nm at room temperature. Despite the fluorescence measurements performed with SOSG is sufficient to assess the production of ^1^O_2_, we also carried out measurements to detect it using an experimental setup like the one proposed by Boso et al. [49]. An HP-120 UV (Opsytec Dr. Gröebel GmbH) point source (120 W), which generates UV and visible radiation, was employed to irradiate the samples. A pair of converging lens guides the beam to a dichroic mirror (DMLP900, Thorlabs), which serves a double function: reflecting the beam to the sample and transmitting the infrared signal coming from the sample towards a set of filters. The first is a longpass filter, and the second is a shortpass filter, with a wavelength of 1200 nm and 1300 nm, respectively (67–296 and 84–642, Edmund Optics). In this way, it is guaranteed that only ^1^O_2_ molecules are recognized. Next, a lens concentrates the infrared radiation into an InGaAs commercial detector (918D-IG-OD1R, Newport), which is connected to a power meter (1918-R, Newport) that produces a power signal when infrared photons are detected. In this experiment, the sample (1 mL) with pheomelanin nanoparticles (2.63 mg/mL) was placed in a 35 mm Petri-dish and exposed to UVA radiation for 3 minutes. The control sample was milli-Q water.

### Statistical analysis

The results are shown as means ± standard deviations, where at least three independent experiments were performed and measured in triplicate. We used the SPSS Software for Windows, version 22 (IBM SPSS Statics, 32 bits). Significance analysis was done with nonparametric Mann-Whitney tests. Significance levels were set at p<0.05.

## Results and discussion

### Dynamic light scattering response

**Fig 2:**
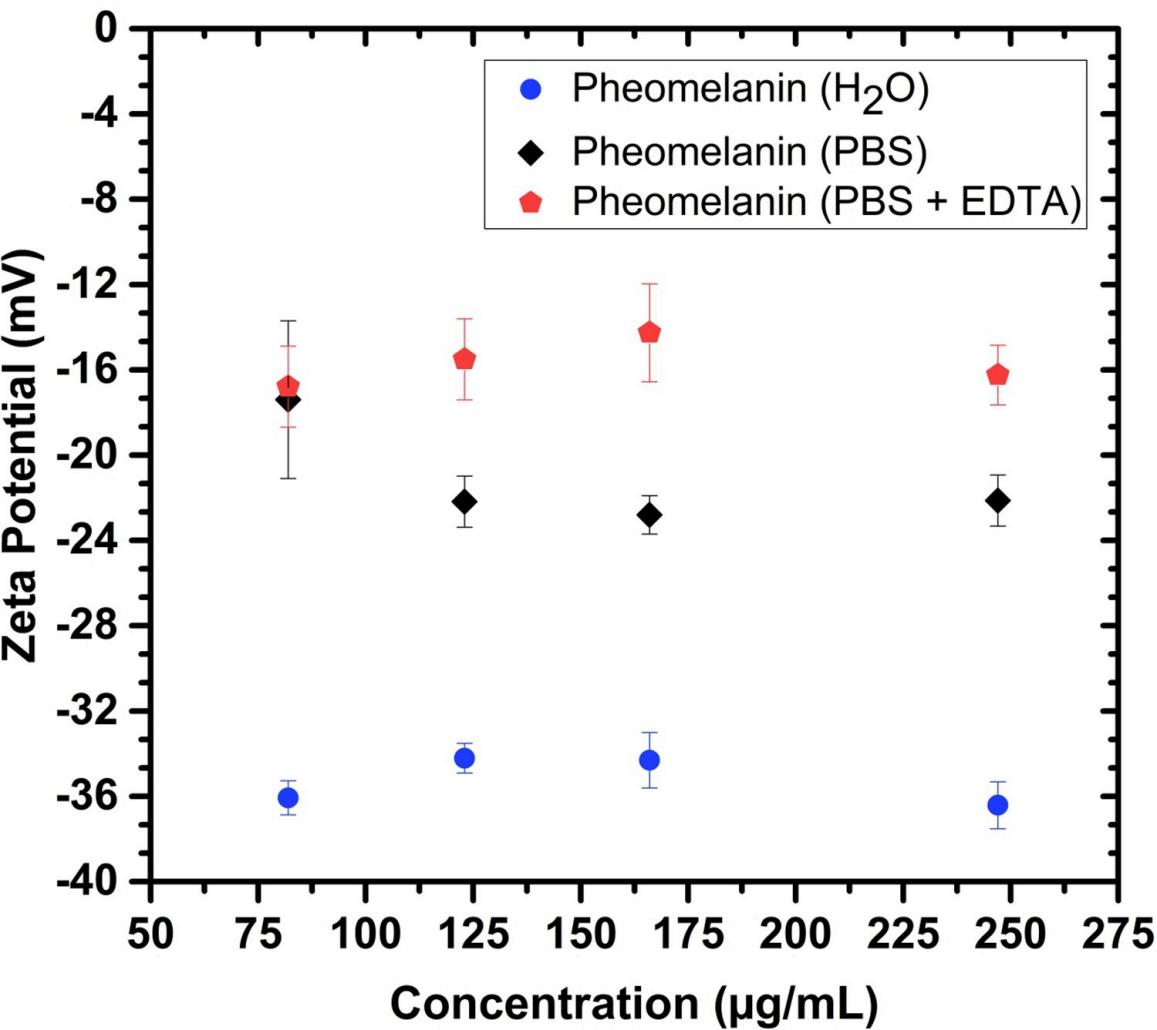
Zeta potential of pheomelanin nanoparticles as a function of concentration. Zeta potential of pheomelanin nanoparticles in different solvents: milli-Q water (blue), PBS (black) and PBS+EDTA (red), at 166 μg/mL.

The zeta potential of pheomelanin particles as a function of concentration is depicted in Fig 2. Note that they are negatively charged, a feature more pronounced in the milli-Q water solvent. At the particle concentration used for most inactivation experiments (166 μg/mL), the zeta potential is approximately -34.4 mV. When the particles are suspended in the buffer used in the *E*. *coli* cultures (PBS), the zeta potential drastically changes to -22.7 mV, indicating a screening effect due to cations dissolved in the medium. Furthermore, the addition of the chelant agent (EDTA) augments the zeta potential to -14.3 mV (enhancing the screening). This result has an impact on the inactivation process, as we will discuss later.

### Scanning electron microscopy measurements

**Fig 3:**
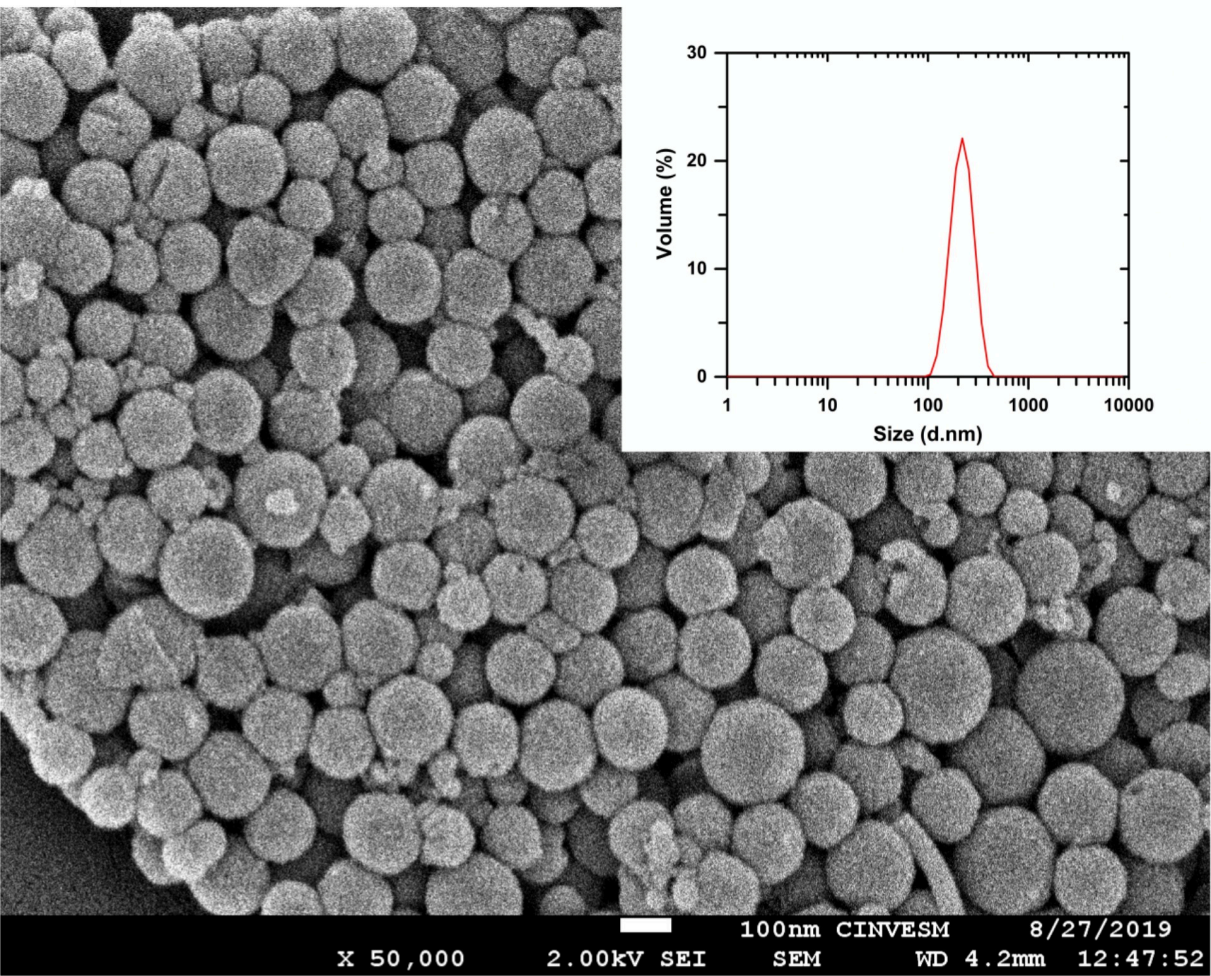
Scanning electron microscopy and particle size distribution. Scanning electron micrograph image of synthesized pheomelanin at 50 k X and the particle size distribution (inset) in PBS (n = 10).

The size and morphology of the pheomelanin nanoparticles are shown in an optimal representative image obtained by SEM, see Fig 3. The nanoparticles have a spherical shape with a diameter around 90–180 nm. The DLS measurements display narrow and unimodal distributions with an average size 200 ± 17 nm (see the inset in the same figure). We include a detailed SEM image with a greater magnification in S3 Fig.

### Toxicity and photoinactivation of pheomelanin nanoparticles

**Fig 4:**
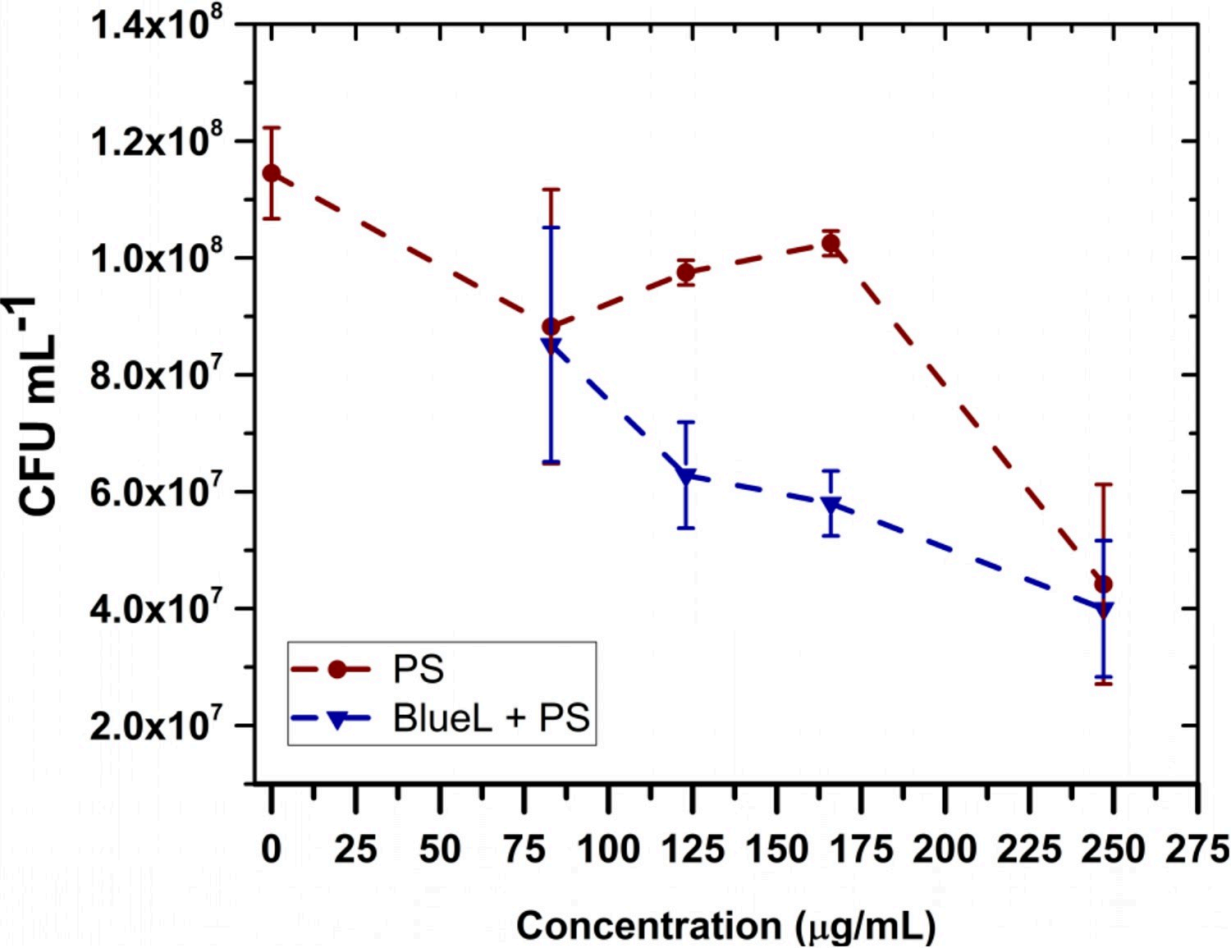
Toxicity and photoinactivation effect of pheomelanin nanoparticles. Dark toxicity (circles) and bactericidal effect (triangles) of pheomelanin nanoparticles at different concentrations against *E*. *coli* exposed 1 h to blue light. Values represent averages ± standard deviations of three different experiments.

We first investigated the dark toxicity and inhibition effect of pheomelanin nanoparticles with blue light (450 nm), at a fixed fluence of 90 J/cm^2^ (1 h). We varied the concentration of PS as shown in Fig 4. The survival of bacteria was determined by counting CFUs. Pheomelanin displayed a low (83, 123 and 166 μg/mL) and high (247 μg/mL) toxicity in *E*. *coli* in dark conditions (see red circles). Since the dark toxicity is around the same in the first three concentrations, we selected 166 μg/mL to perform the inactivation experiments.

At this concentration, the dark toxicity was 9.5% and the inactivation efficacy with this fluence (90 J/cm^2^) was 48.7%. Next, we explored the photoinactivation effect for other fluences. Tables 1 and 2 show the reduction of CFU for 180 and 270 J/cm^2^ (2 and 3 h) using blue and UVA radiation. In the first case, the photoinactivation was 61 and 79%, respectively. Meanwhile, for UVA it was 91 and 99%, respectively.

**Table 1:** Effect of blue light on *E*. *coli* using pheomelanin nanoparticles.

Sample	CFU (log10) mL^-1^	
	2 h	3 h
PBS	8.05 ± 0.05	7.99 ± 0.10
PS	7.88 ± 0.04	7.82 ± 0.09
Blue Light	- ^1^	8.16 ± 0.03
PS + Blue Light	7.64 ± 0.23	7.31 ± 0.22

^1^ Not measured since it was considered only the highest fluence (3 h).

**Table 2:** Effect of UVA radiation on *E*. *coli* using pheomelanin nanoparticles.

Sample	CFU (log10) mL^-1^	
	2 h	3 h
PBS	8.02 ± 0.12	8.18 ± 0.18
PS	7.85 ± 0.05	7.57 ± 0.29
UVA	- ^1^	7.89 ± 0.06
PS + UVA	6.95 ± 0.23	6.04 ± 0.55

^1^ Not measured since it was considered only the highest fluence (3 h).

Thereafter, we explored the inactivation response enhanced by the chelant agent EDTA. For this purpose, we decided to use the highest fluence for both, blue and UVA light.

**Fig 5:**
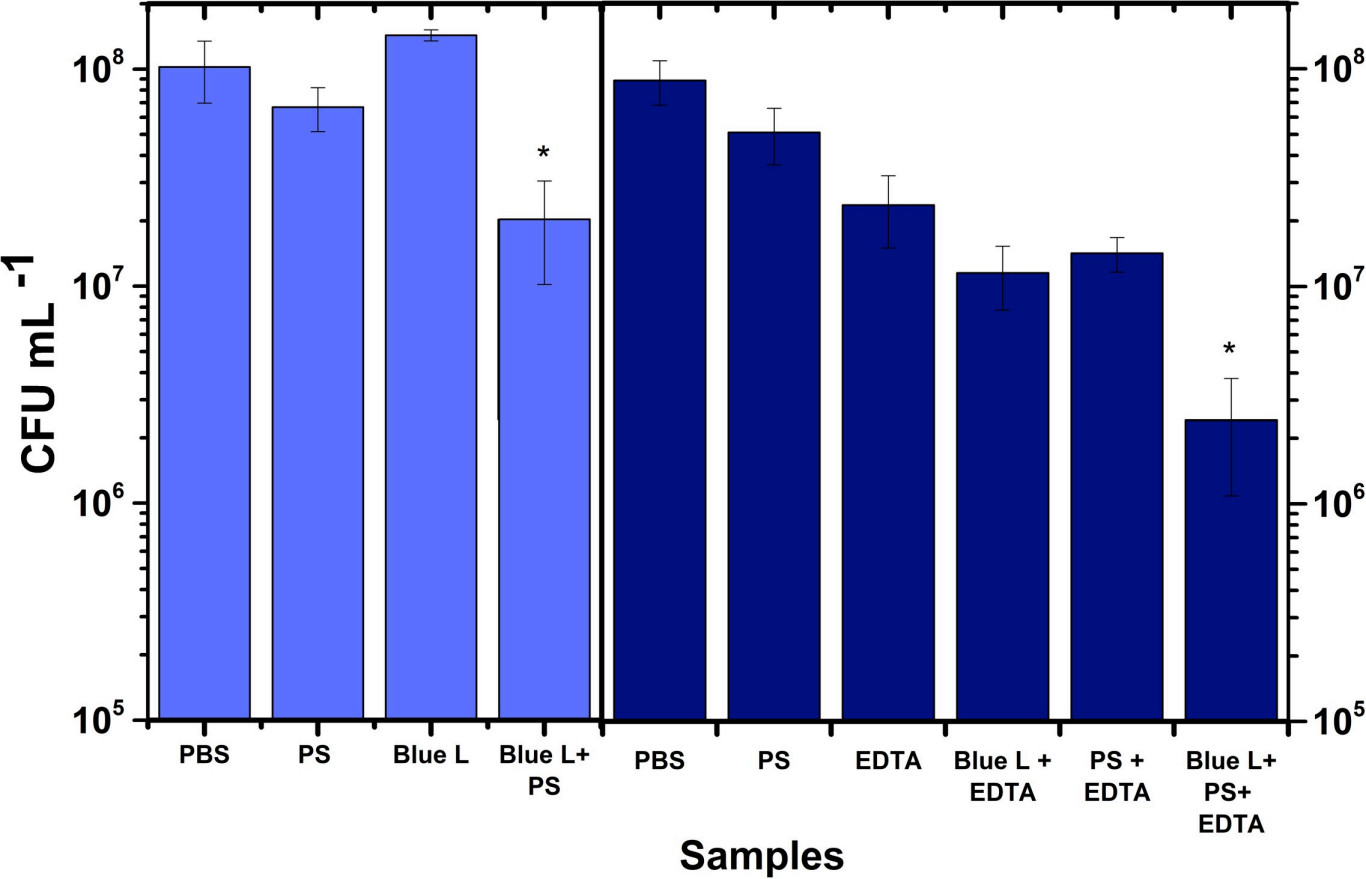
*E*. *coli* photoinactivation with pheomelanin nanoparticles excited with blue light with and without EDTA (10 mM). The cultures at 166 μg/mL of pheomelanin nanoparticles were irradiated during 3 h (270 J/cm^2^). Controls are PBS, PS, Blue light and EDTA. The strongest photoinactivation effect is depicted as Blue L+PS+EDTA. Values are averages and standard deviations of three biological triplicate. * p<0.05.

Fig 5 displays the results of photoinactivation with blue light in the absence and presence of EDTA. Note that there are two PBS and PS controls. The reason is because the group to assess the effect of blue light was different from the group to evaluate the effect of EDTA. Clearly, blue light by itself does not produce photoinactivation. It can be observed that the viability of bacteria decreased approximately 80% after irradiation with blue light. The addition of EDTA showed further inhibitory effect (97%). Altogether, the illumination of these cultures with blue light leads to a reduction in bacterial population of 1.5 log10 steps CFU.

**Fig 6:**
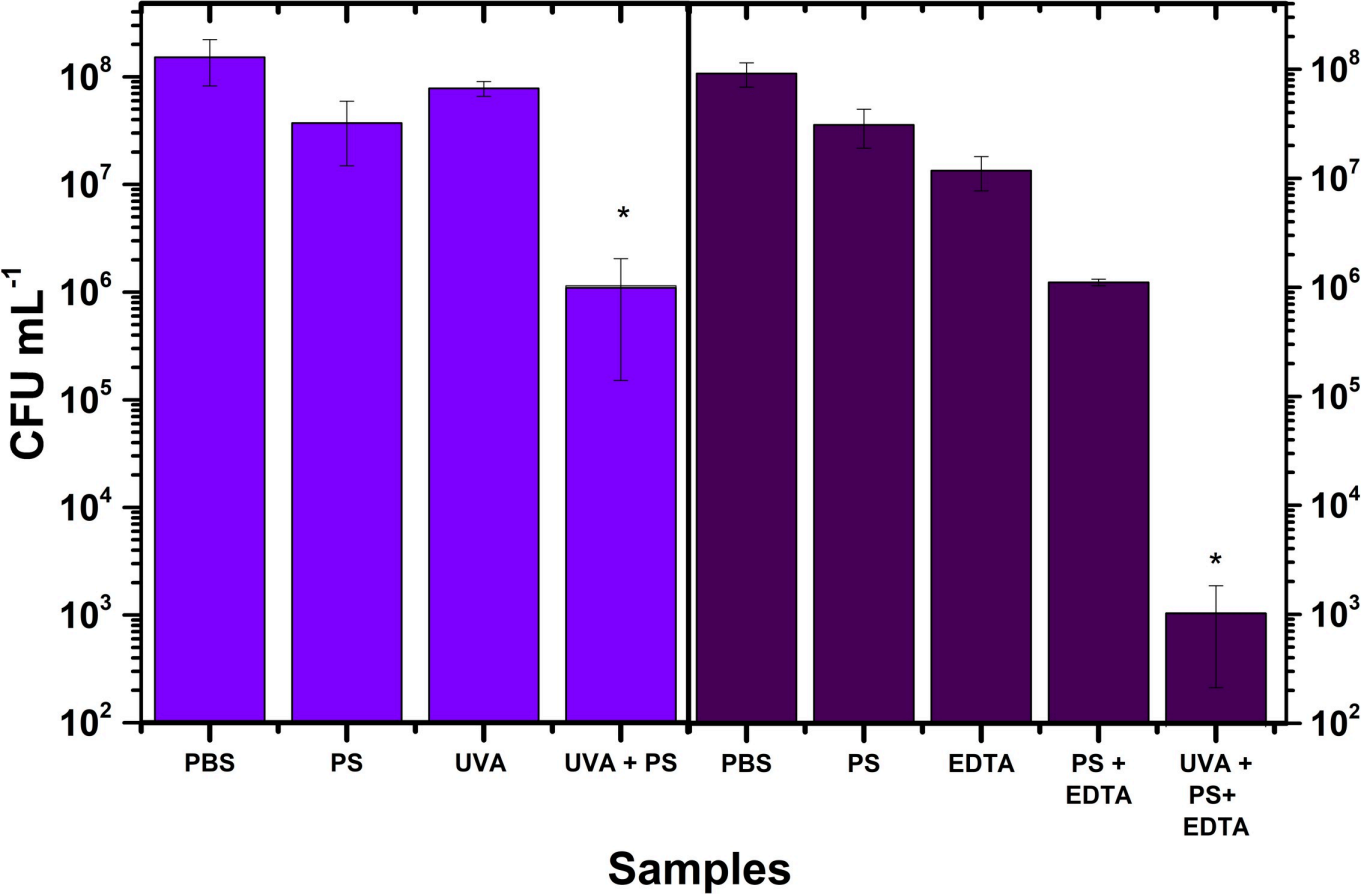
*E*. *coli* photoinactivation with pheomelanin nanoparticles excited with UVA radiation with and without EDTA (10 mM). The cultures at 166 μg/mL of pheomelanin nanoparticles were irradiated during 3 h (270 J/cm^2^). Controls are PBS, PS, UVA and EDTA. The strongest photoinactivation effect is depicted as UVA+PS+EDTA. Values are averages and standard deviations of three biological triplicate. * p<0.05.

Similarly, the inactivation effect of UVA radiation is displayed in Fig 6. Be aware that there are two PBS and PS controls. As before, the reason is because the group to assess the effect of UVA radiation was different from the group to evaluate the effect of EDTA. We would like to remark that UVA alone does not produce photoinactivation. As shown, the reduction in the CFU was 2 log10 steps, whereas the use of EDTA resulted in a reduction of 5 log10.

### Fluorescence assays to detect ROS

**Fig 7:**
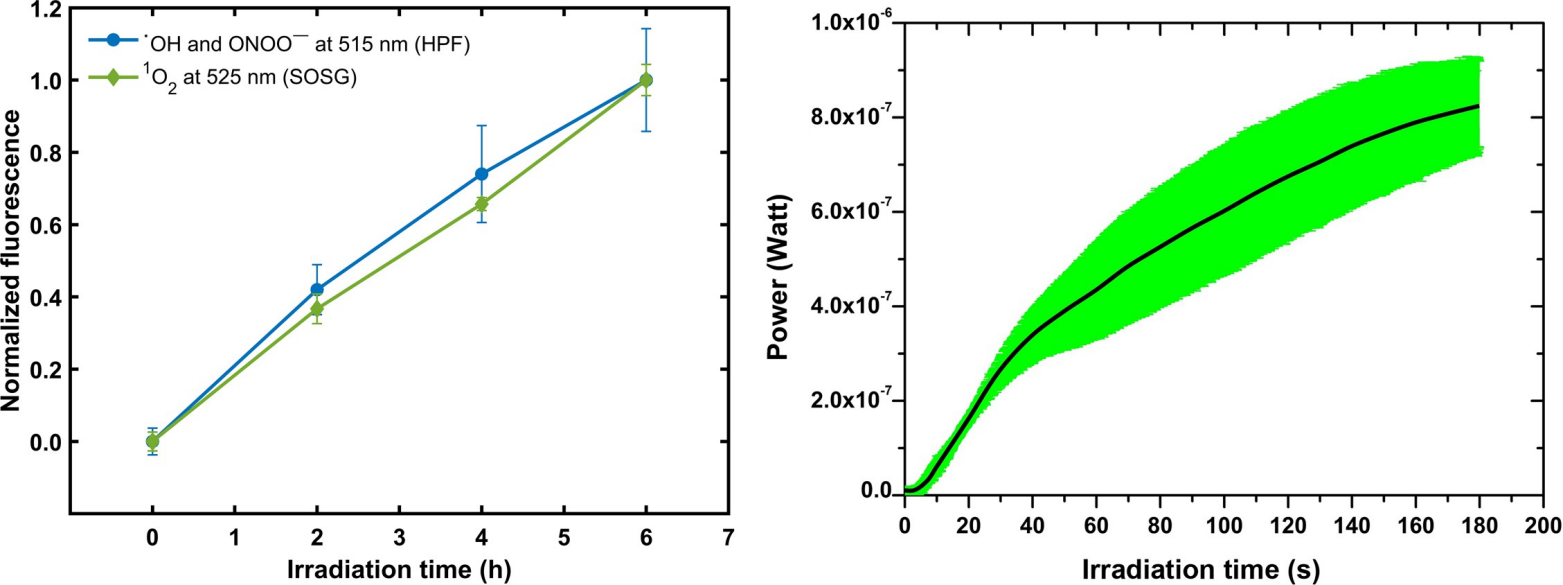
ROS assessment. (A) Fluorescence generated from SOSG (1.92 μM) and HPF (5 mM**)** with pheomelanin nanoparticles, at 525 (green diamonds) and 515 nm (blue circles), respectively. Data are normalized to the maximum value in each case: 3.44x10^6^, and 3.42x10^6^ fluorescence units for SOSG and HPF, respectively. The samples were exposed to UVA radiation during 6 hours and evaluated every 2 hours. (B) Power emitted by singlet oxygen during UVA radiation (see section Materials and Methods). The measurements were obtained for 3 minutes. The black line is the average and the band the dispersion of the data from three independent experiments. The large dispersion is due to the detection method.

Fig 7A depicts the fluorescence intensity from ROS generated by pheomelanin nanoparticles with two different sensors: SOSG (diamonds) and HPF (circles), exposed to UVA radiation. In both cases, the fluorescence intensity increases as the irradiation time augments. For the detection of ^1^O_2_, we also carried out a second measuring method using a solid-state detector (InGaAs) [49]. Fig 7B shows the increment of the power intensity in real-time as the pheomelanin suspension is irradiated, indicating the immediate generation of singlet oxygen. When the rate of generation is maximum, the power saturates rapidly (in around three minutes).

### Final remarks

Although some properties and effects caused by pheomelanin nanoparticles have been investigated in previous works [1, 25, 38], there is not reported evidence about its inactivation action in bacteria. Due to the fact *E*. *coli* is Gram-negative, its photoinactivation is a challenging aim. As we highlighted previously, pheomelanin particles are negatively charged (see Fig 2). Hence, they are unable to approach bacteria (which carry also negative charges) hindering its inactivation effect. We have learnt here that the chelant agent EDTA not only modifies the permeability of the cellular membranes but also reduces the zeta potential of the particles as observed in Fig 2. This can be explained by a screening effect on the particles, which is enhanced by the fact EDTA is a weak acid (it protonates at the pH of the solution regulated by PBS). Thus, the electrostatic repulsion with bacteria is diminished, improving the photoinactivation action. We would like to emphasize that we are not the first to employ EDTA with this purpose; other research groups have also used it in conjunction with different non cationic photosensitizers to enhance the effect against Gram-negative bacteria [40–42].

Because photoinactivation is associated to the generation of ROS, we carried out measurements to assess the production of hydroxyl radicals, peroxynitrates, and ^1^O_2_ generated by pheomelanin nanoparticles exposed to UVA radiation. It has been reported that pheomelanin exposed to UVA leads to an oxidative damage in cellular components, which is mainly due to peroxides and hydroxyl radical [50, 51], both assessed in this work as well as singlet oxygen (see Fig 7A and 7B).

It could be thought that the irradiation time employed in the present photoinactivation experiments may not be so practical. However, let us note that such large times are not unusual as reported in other photoinactivation studies [52–56].

## Conclusions

In this work, we investigated for the first time the photoinactivation of *E*. *coli* bacteria using pheomelanin nanoparticles. Since pheomelanin is not commercially available, we synthesized good quality nanoparticles by an oxidative process previously proposed by Pyo et al. [25] with some pertinent modifications. Several characterization techniques were performed to confirm the nature of our pheomelanin nanoparticles. We found that EDTA not only modifies the permeability of the cellular membrane but almost neutralizes the electrical charge of such nanoparticles, enhancing bacterial photoinactivation. Our experiments were carried out using blue and UVA light. The most significant inactivations were achieved with UVA radiation at a particle concentration of 166 μg/mL and fluence of 270 J/cm^2^. In summary, the photoinactivation procedure with the mixture of pheomelanin nanoparticles and EDTA was able to reduce up to 5 log10 with respect to the PBS control.

## Supporting information

S1 File Synthesis and characterization. (PDF)

S1 Fig Absorbance spectra of pheomelanin (red) and eumelanin (brown). The correct synthesis of the pigment was confirmed by UV-Vis spectroscopy. Indeed, the process was monitored through the evolution of the absorbance spectrum until the absorbance peak of L-DOPA disappears (around 12 h). It is shown the UV-Vis absorbance spectrum of the synthesized pheomelanin and commercial eumelanin (M8631, Sigma-Aldrich) showing good similarity between the two pigments. Both exhibit a strong absorption in the region 300–450 nm, with a monotonic decay behaviour that extended to the infrared region. In general, pheomelanin showed a higher absorption in the full spectrum, becoming more significant in the visible region, according to those reported by Pyo et al. [25].
(JPG)

S2 Fig Scanning electron micrograph image of synthesized pheomelanin nanoparticles at 100 kX magnification. Although there is a slight polydispersity, the size of the particles does not surpass 200 nm.
(PDF)

S3 Fig X-ray diffraction of synthesized pheomelanin powder showing its amorphous features. The XRD spectrum of pheomelanin nanoparticles gives a broad diffraction peak centered approximately at 2θ = 25°. It is well known that such peak is distinctive of amorphous and disordered compounds. The scattering of X-rays is non-coherent since this structure does not show a continuous and organized pattern, which is a classic feature of melanins [27–30]. On the contrary, sharp peaks are displayed by crystalline compounds. Note that the spectra are quite similar, therefore we used two other techniques to gather more information about the sulphur signal.
(JPG)

S4 Fig Fourier transform infrared spectrum of pheomelanin powder in different regions compared with eumelanin powder (inset). A representative FTIR spectrum of pheomelanin nanoparticles and synthetic eumelanin. Note that both of them exhibit the dominant peak of water around 3700 cm^-1^, coming from the strong hydroxyl stretching vibrations (OH_ν_), as well as the sharp and weaker hydroxyl bending mode (OH_δ_) around 1600 cm^-1^ [33], and the CH deformation band at 1280 cm^-1^ [34]. In contrast and as expected, the pheomelanin sample reveals the distinctive presence of sulphur. Indeed, it is observed the characteristic transmission band C-S_ν_ around 685 cm^-1^, and a signal for aromatic rings (C-H of C = C-H) at 800 cm^-1^, according to a previous report [35]. The weak peaks at approximately 1280 and 1213 cm^-1^ are characteristic for pheomelanin, which corresponds to (COH) phenolic stretching and S-O, respectively. In the case of eumelanin, a prominent distinctive peak is displayed around 1710 cm^-1^ for C = O stretching in COOH [24, 36, 37].
(JPG)

S1 Table Summary of the percentage contributions of the main detected elements from seven samples of pheomelanin. The results for eumelanin are also displayed for comparative purposes. The nature of the synthetized sample was confirmed by a percentage elemental analysis showing 4% of sulphur, which is not present in eumelanins (commercial and synthesized). Note that there are also appreciable differences in the elements C, H and O, in agreement with previous results [27, 31]. Additionally, small traces (less than 1%) of Mn were detected in some samples. Although oxidation has been observed in some alcohols containing MnO_2_ after irradiation with blue light, the amount of this compound was high [32]. In our experimental conditions, the amount of Mn is not relevant for bacterial activity.
(DOCX)
