# Supplementary material for: The growth of Escherichia coli cultures under the influence of pheomelanin nanoparticles and a chelant agent in the presence of light
Source: PLoS One. 2022 Mar 11;17(3):e0265277. doi: 10.1371/journal.pone.0265277 (PMC8916617; doi:10.1371/journal.pone.0265277)
Supplement: S1 File — (PDF) [file pone.0265277.s001.pdf]

The growth of *Escherichia coli* cultures under the influence of pheomelanin nanoparticles and a chelant agent in the presence of light

Denisse Fuentes-López[1], Daniel Ortega-Zambrano[1], María Antonieta Fernández-Herrera[2], Hilda Mercado-Uribe[1,*]

[1] Centro de Investigación y de Estudios Avanzados
del Instituto Politécnico Nacional Unidad Monterrey
PIIT, Autopista al Aeropuerto, km. 9.5
Apodaca, Nuevo León, 66600, México

[2] Departamento de Física Aplicada
Centro de Investigación y de Estudios Avanzados
del Instituto Politécnico Nacional Unidad Mérida, km. 6
Antigua Carretera a Progreso
Apdo. Postal 73, Cordemex
Mérida, Yucatán, 97310, México

[*] Corresponding author
Hilda Mercado-Uribe (HM)
E-mail: hmercado@cinvestav.mx
ORCID: 0000-0002-1123-7375

Supporting information

Based on the oxidation method proposed by Pyo et al. [1] we present below the details of the pheomelanin nanoparticles synthesis used in our research work. We also show the results of the characterization with different methods.

Synthesis

For the synthesis of pheomelanin particles, 70 mg (0.35 mmol) of 3,4-dihydroxyL-phenylalanine (L-DOPA, D9628, Sigma-Aldrich) was dissolved in deionized water (milli-Q, 18.2 MΩcm) at 50 ^{o}C. The solution was then brought to room temperature. Next, 85.6 mg (0.70 mmol) of L-Cysteine (C7352, Sigma-Aldrich) was added to the solution followed by 360 µL of $KMnO_4$ (1 N, 223468, Sigma-Aldrich). The referred process was carried out under continuous stirring at 1500 r.p.m during 24 h and dark conditions. The obtained particles were precipitated by centrifugation for 10 min at 20,000 r.p.m. They were resuspended in 50 mL of deionized water and mixed with 1 mL of HCl (1 M) to exchange the Mn^{2+} ions in the solution. In order to raise the pH of the sample, the suspension was subjected to six washing cycles at the formerly described conditions. A last centrifugation (10 min at 4500 r.p.m) was carried out to discard big aggregates. The final value of pH was 6. The resulting nanoparticles were resuspended in PBS in order to obtain a stable medium for the cell culture. The solution, now with pH 7.4 was stored in an amber glass jar at 4 ^{o}C.

UV-Vis spectroscopy

The synthesis process was monitored by assessing the change of the maximum absorbance peak from the precursor L-DOPA (at 280 nm) using a spectrophotometer (Multiskan, GO Thermo Scientific). Once the pheomelanin particles were obtained, 1 mL of the solution was poured into a polystyrene cell and the absorbance spectrum from 200 to 1000 nm was obtained.

Lyophilization

Pheomelanin solution was lyophilized using a Labconco FreeZone 2.5 system. The solution was first centrifuged at 20,000 r.p.m to remove water excess. The samples were liophylized at a pressure and temperature < 0.133 mBa and -40 ^oC and stored at -20 ^oC between 1 and 3 days before characterization.

Characterization methods

Dynamic light scattering

Particle sizes and zeta potentials were determined by dynamic light scattering (DLS) using a Malvern Zetasizer NanoZS equipment**.** See main text for details.

Fourier transform infrared spectroscopy

The functional groups present in the synthesized samples allow us to assess the purity of the pheomelanin particles. The samples were evaluated using a FTIR spectrometer (Agilent Cary 630) in the transmittance mode. The powder was placed on the ATR accessory (DATR Agilent) made of diamond crystal (R.I=2.4). The spectrum was obtained in the range 4000-650 cm^{-1}, at a path length of 2.6 µm and room temperature. A total of 32 scans were performed with step of 4 cm^{-1}. Every experiment was carried out ten times using different samples. All spectra were background subtracted and smoothed with 7-point Savitsky-Golay function to reduce the noise. The data were analyzed using the Origin 2016 software.

X-ray diffraction analysis

X ray diffraction (XRD) patterns of the samples were obtained on a XRD system (Bruker D-8 Advance) using CuKα radiation. The instrument was operated at 30 mA and 40 kV. The scans were performed between 3-60 ^{o} 2θ, using a step size of 0.02 and dwell time of 0.5 s. The experiment was performed seven times. Data acquisition and evaluation were performed by the Diffract.suite software package.
