# Supplementary figures and images for: The growth of Escherichia coli cultures under the influence of pheomelanin nanoparticles and a chelant agent in the presence of light
Source: PLoS One. 2022 Mar 11;17(3):e0265277. doi: 10.1371/journal.pone.0265277 (PMC8916617; doi:10.1371/journal.pone.0265277)

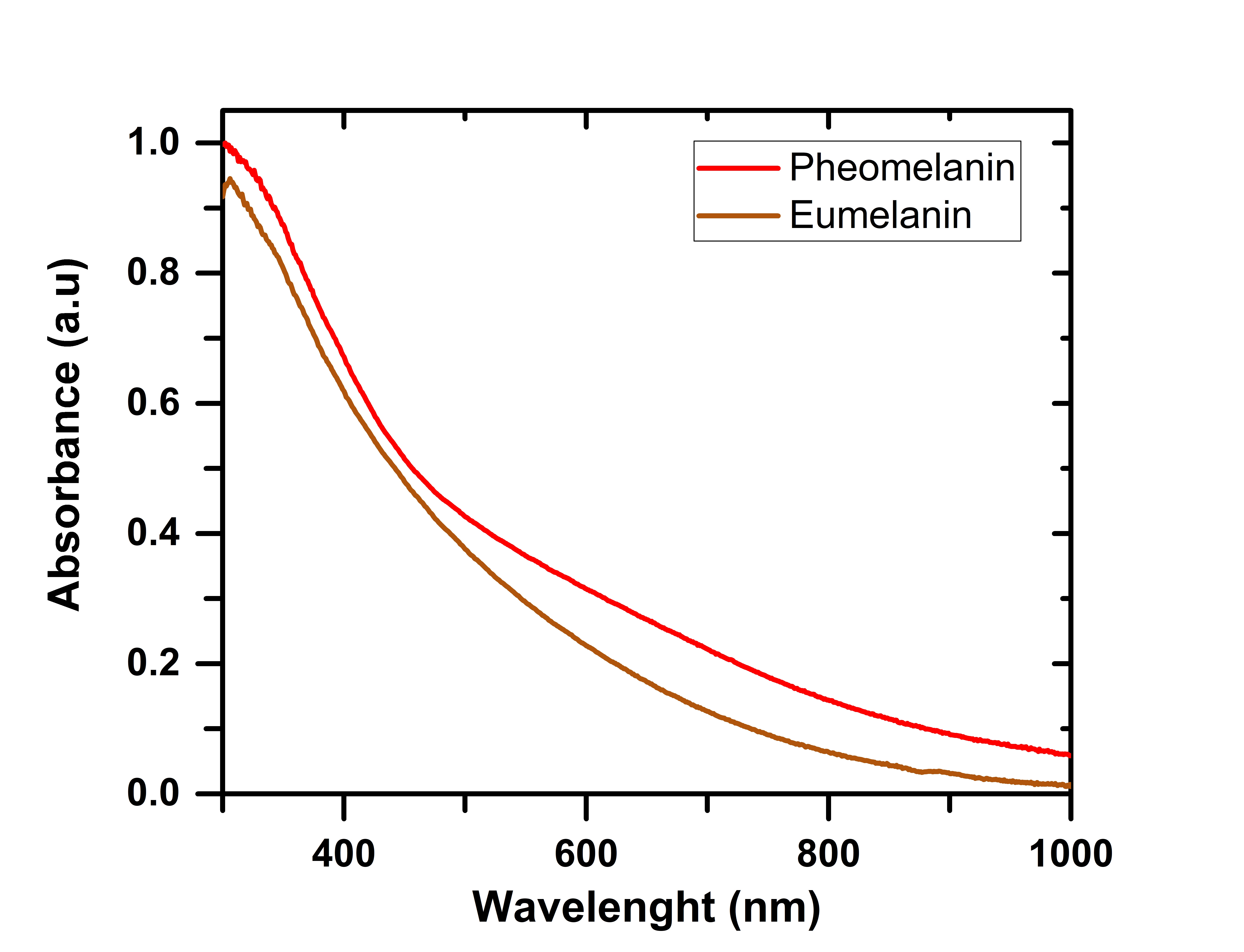

Supplement: S1 Fig — The correct synthesis of the pigment was confirmed by UV-Vis spectroscopy. Indeed, the process was monitored through the evolution of the absorbance spectrum until the absorbance peak of L-DOPA disappears (around 12 h). It is shown the UV-Vis absorbance spectrum of the synthesized pheomelanin and commercial eumelanin (M8631, Sigma-Aldrich) showing good similarity between the two pigments. Both exhibit a strong absorption in the region 300–450 nm, with a monotonic decay behaviour that extended to the infrared region. In general, pheomelanin showed a higher absorption in the full spectrum, becoming more significant in the visible region, according to those reported by Pyo et al. [25]. (JPG) [file pone.0265277.s002.JPG]

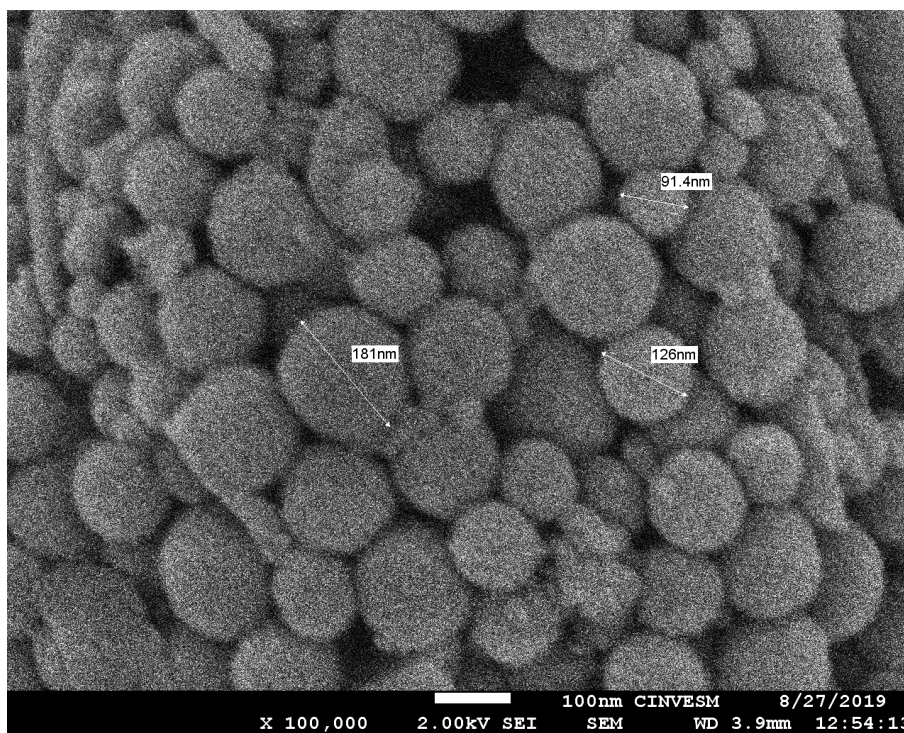

Supplement: S2 Fig — Although there is a slight polydispersity, the size of the particles does not surpass 200 nm. (PDF) [file pone.0265277.s003.pdf]

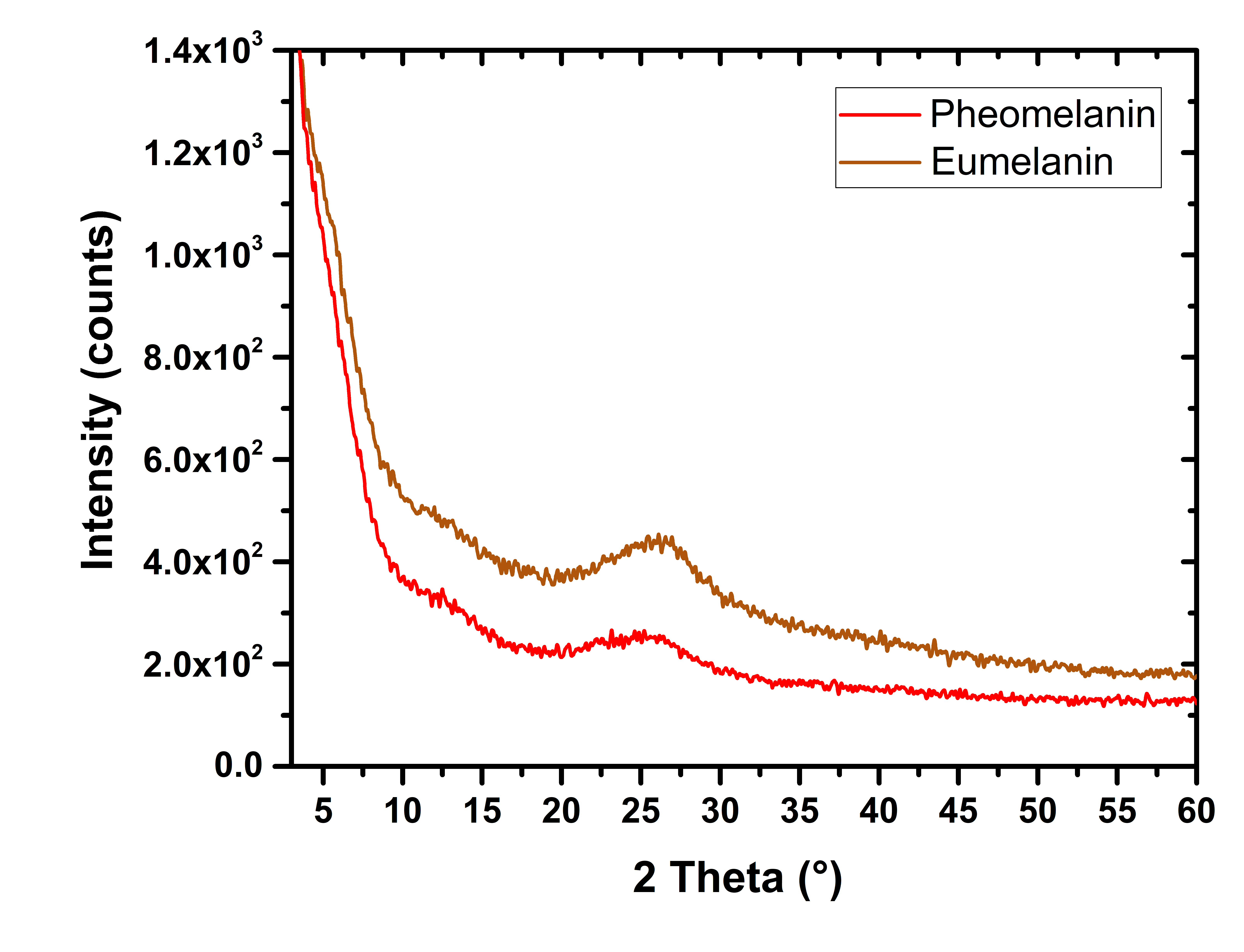

Supplement: S3 Fig — The XRD spectrum of pheomelanin nanoparticles gives a broad diffraction peak centered approximately at 2θ = 25°. It is well known that such peak is distinctive of amorphous and disordered compounds. The scattering of X-rays is non-coherent since this structure does not show a continuous and organized pattern, which is a classic feature of melanins [27–30]. On the contrary, sharp peaks are displayed by crystalline compounds. Note that the spectra are quite similar, therefore we used two other techniques to gather more information about the sulphur signal. (JPG) [file pone.0265277.s004.JPG]

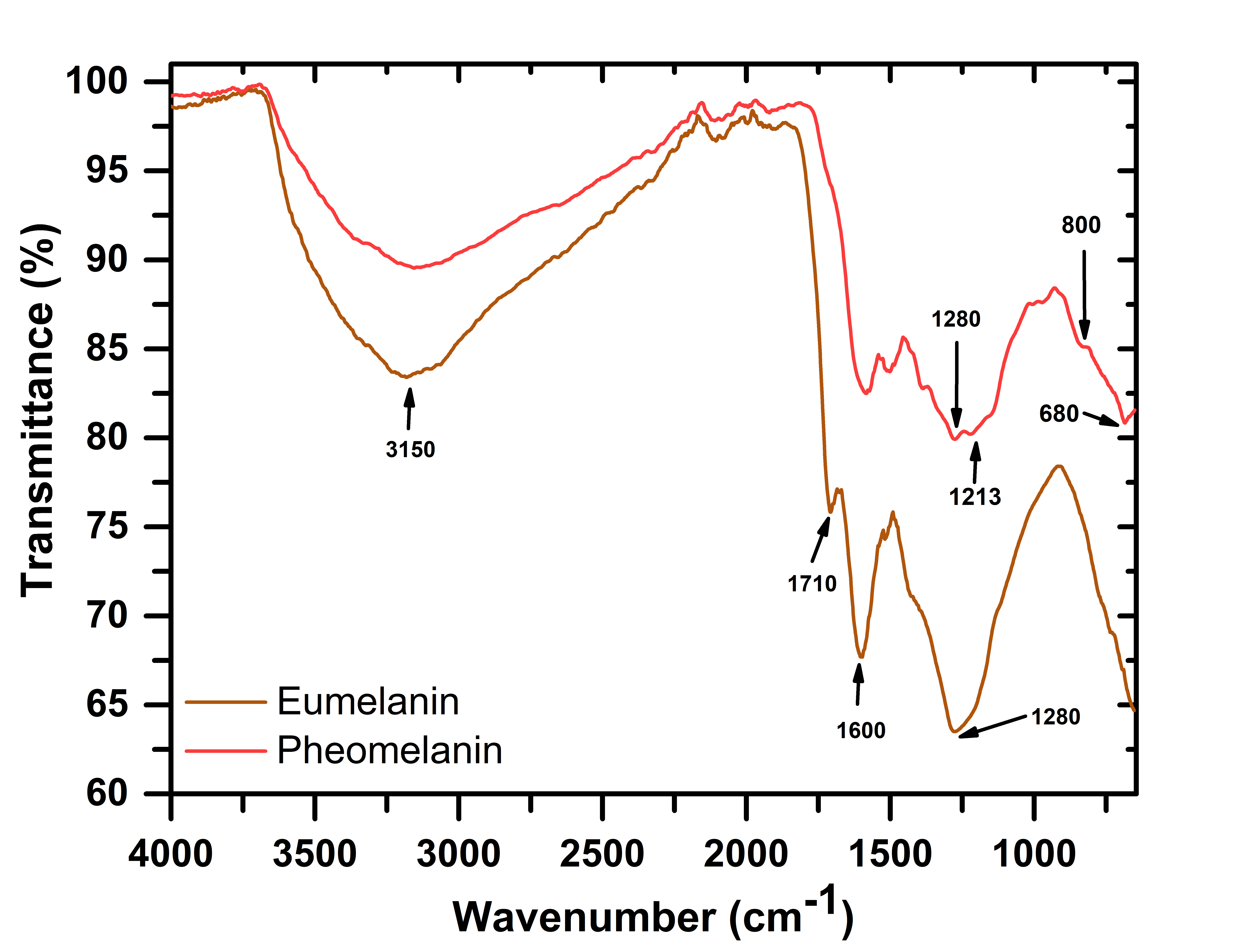

Supplement: S4 Fig — A representative FTIR spectrum of pheomelanin nanoparticles and synthetic eumelanin. Note that both of them exhibit the dominant peak of water around 3700 cm-1, coming from the strong hydroxyl stretching vibrations (OHν), as well as the sharp and weaker hydroxyl bending mode (OHδ) around 1600 cm-1 [33], and the CH deformation band at 1280 cm-1 [34]. In contrast and as expected, the pheomelanin sample reveals the distinctive presence of sulphur. Indeed, it is observed the characteristic transmission band C-Sν around 685 cm-1, and a signal for aromatic rings (C-H of C = C-H) at 800 cm-1, according to a previous report [35]. The weak peaks at approximately 1280 and 1213 cm-1 are characteristic for pheomelanin, which corresponds to (COH) phenolic stretching and S-O, respectively. In the case of eumelanin, a prominent distinctive peak is displayed around 1710 cm-1 for C = O stretching in COOH [24, 36, 37]. (JPG) [file pone.0265277.s005.JPG]
