# Supplementary material for: The growth of Escherichia coli cultures under the influence of pheomelanin nanoparticles and a chelant agent in the presence of light
Source: PLoS One. 2022 Mar 11;17(3):e0265277. doi: 10.1371/journal.pone.0265277 (PMC8916617; doi:10.1371/journal.pone.0265277)
Supplement: S1 Table — The results for eumelanin are also displayed for comparative purposes. The nature of the synthetized sample was confirmed by a percentage elemental analysis showing 4% of sulphur, which is not present in eumelanins (commercial and synthesized). Note that there are also appreciable differences in the elements C, H and O, in agreement with previous results [27, 31]. Additionally, small traces (less than 1%) of Mn were detected in some samples. Although oxidation has been observed in some alcohols containing MnO2 after irradiation with blue light, the amount of this compound was high [32]. In our experimental conditions, the amount of Mn is not relevant for bacterial activity. (DOCX) [file pone.0265277.s006.docx]

**S5 Table 1. Summary of the percentage contributions of the main detected elements from seven samples of pheomelanin. The results for eumelanin are also displayed for comparative purposes.**

| Sample | C (wt%) | N (wt%) | O (wt%) | S (wt%) |
| --- | --- | --- | --- | --- |
| Eumelanin | 49.53 | 10.31 | 40.16 | 0 |
| PM 1 | 56.87 | 12.15 | 27.19 | 3.78 |
| PM 2 | 51.76 | 13.15 | 31.39 | 3.7 |
| PM 3 | 55.69 | 10.03 | 29.83 | 4.45 |
| PM 4 | 47.22 | 12.24 | 34.98 | 5.55 |
| PM 5 | 50.18 | 14.41 | 31.33 | 4.08 |
| PM 6 | 50.47 | 12.47 | 32.41 | 4.66 |
| PM 7 | 50.86 | 12.47 | 32.39 | 4.28 |
| PM average | 51.86 | 12.42 | 31.36 | 4.36 |
| PM SD | 3.34 | 1.31 | 2.42 | 0.66 |
